# Exploring the Antimicrobial Stewardship Educational Needs of Healthcare Students and the Potential of an Antimicrobial Prescribing App as an Educational Tool in Selected African Countries

**DOI:** 10.3390/antibiotics11050691

**Published:** 2022-05-19

**Authors:** Omotola Ogunnigbo, Maxencia Nabiryo, Moses Atteh, Eric Muringu, Olatunde James Olaitan, Victoria Rutter, Diane Ashiru-Oredope

**Affiliations:** 1 Commonwealth Pharmacists Association, London E1W 1AW, UK; omotola.ogunnigbo@commonwealthpharmacy.org (O.O.); maxencia.nabiryo@commonwealthpharmacy.org (M.N.); victoria.rutter@commonwealthpharmacy.org (V.R.); 2 Grand Erie District School Board, Brantford, ON N3T 5V3, Canada; moses.atteh@granderie.ca; 3 Pharmaceutical Society of Kenya, Hurlingham, Jabavu Lane, Nairobi P.O. Box 44290-00100, Kenya; emuringu158@gmail.com; 4 Department of Pharmaceutical and Medicinal Chemistry, Faculty of Pharmacy, Olabisi Onabanjo University, Ago Iwoye P.O. Box 2002, Nigeria; olatunde.olaitan@oouagoiwoye.edu.ng

**Keywords:** antimicrobial resistance, CwPAMS, prescribing app, antimicrobial surveillance, antibiotics, antimicrobials, antimicrobial stewardship

## Abstract

Antimicrobial resistance (AMR) is a global health threat and one of the top 10 global public health threats facing humanity. AMR contributes to 700,000 deaths annually and more deaths, as many as 10 million are projected to happen by 2050. Antimicrobial stewardship (AMS) activities have been important in combating the ripple effects of AMR and several concerted efforts have been taken to address the issues of antimicrobial resistance. The Commonwealth Pharmacists Association through the Commonwealth Partnerships for Antimicrobial Stewardship (CwPAMS) programme has been enhancing the capacity of health institutions in Low-Middle-Income Countries (LMIC) to combat AMR. Through such efforts, an antimicrobial prescribing app (CwPAMS app) was launched and delivered to support antimicrobial prescribing and improve AMS practice in four African countries; Ghana, Uganda, Zambia, and Tanzania. The app provides easy access to infection management resources to improve appropriate use of antimicrobials in line with national and international guidelines. This study aimed to identify and explore the potential for the usability of the CwPAMS app among healthcare students across selected African countries that are part of the Commonwealth. The study equally evaluated the healthcare students’ understanding and attitudes towards antimicrobial resistance and stewardship. Despite 70% of the respondents indicating that they had been taught about prudent use of antibiotics, diagnosis of infections and their management using antibiotics in their universities, notable knowledge gaps were discovered: 52.2% of the respondents had no prior information on the term AMS, 50.6% of them reported a lack of resources for accessing up-to-date information on drugs, for instance only 36% had had an opportunity to access an app as a learning resource even when 70% of the respondents thought that a mobile app would support in increasing their knowledge. Those challenges reveal an opportunity for the CwPAMS App as a potential option to address AMR and AMS gaps among healthcare students.

## 1. Introduction

Resistance to antimicrobials threatens the effective prevention and treatment of an ever-increasing range of infections [1]. Antimicrobial resistance is a current global health and development threat and the World Health Organization declared it as one of the top 10 global public health threats facing humanity [1]. The overarching issue is that antimicrobial resistance threatens the very core of modern medicine and the sustainability of an effective global public health response [2]. Each year, 700,000 people lose their lives to AMR, and more deaths.As many as 10 million deaths are projected to happen by 2050 with a 3.8 percent reduction in the annual gross domestic product (GDP) if action is not taken [3]. AMR significantly affects the economy through prolonging illness, thus causing longer hospital stays and the need for more expensive medicines, which pose financial challenges for the national health systems and the affected individuals [1,4].

Whilst resistance to existing antibiotics is increasing, the development of new antibiotics is low. In 2019, the WHO identified only six antibiotics of the 32 antibiotics that were in clinical development that address the WHO list of priority pathogens [1]. Misuse and overuse of antimicrobials are the main drivers in the development of drug-resistant pathogens [1]. Existing literature commonly highlights that health care providers especially those in LMIC have inadequate knowledge on use of antimicrobials and antimicrobial resistance [5,6]. As a result, the challenge of inappropriate prescription of antimicrobials continues to prevail, thus worsening antimicrobial resistance [5,6,7].

Antimicrobial stewardship alongside infection prevention and control have been the mainstay in tackling the challenge of AMR. As a global response, antimicrobial stewardship, which is the systematic, coordinated effort that promotes the appropriate use of antimicrobials was launched through several collaborations and concerted efforts of governments and healthcare bodies and organisations [8,9]. It is important that prescribers ensure “the right antibiotic, at the right dose at the right time” [10] Antimicrobial stewardship programmes should take into consideration monitoring and evaluation of antimicrobial prescribing, regular feedback to prescribers, education and training for health and social care staff, and integrating audits into existing quality improvement programmes [11].

The UK aid Fleming Fund funded a new programme in 2018, the Commonwealth Partnerships for Antimicrobial Stewardship programme (CwPAMS), managed by the Commonwealth Pharmacists Association (CPA) and Tropical Health and Education Trust (THET) and supports improvement of antimicrobial prescribing, surveillance and stewardship [12]. As part of the CwPAMS antimicrobial stewardship interventions, an Antimicrobial Prescribing app (CwPAMS app) was launched in 2019 and delivered to improve antimicrobial prescribing practice in four African countries; Ghana, Uganda, Zambia, and Tanzania [13]. The app provides easy access to infection management resources and improves the appropriate use of antimicrobials according to national and international guidelines.

Following the launch of the CwPAMS app in the four African countries, there were 530 downloads of the app and 2795 guides opened within 12 months. Ghana received more downloads (50.3 percent) than Uganda (31 percent), Tanzania (13 percent), Zambia (1.9 percent), and others (3.8 percent) [13]. Relatedly, Olaoye et al. [13] reported that the CwPAMS app improved the health care providers’ awareness on AMS and prescribing practices. Further, the health care providers found the CwPAMS app easily accessible as its usability is not internet dependent [13]. To respond to one of the recommendations of this study, which was further dissemination of the CwPAMS app; identifying and addressing the educational needs of health care students (future healthcare professionals) was considered paramount to effecting long term and sustainable changes in antimicrobial stewardship. By taking stewardship activities to the grassroots, i.e., healthcare students, they can be better informed on antimicrobial prescribing and more empowered healthcare providers as professionals. The importance of smartphones in healthcare training and education cannot be underestimated. Smartphones are described as a resource called “Learn Anywhere” [14].

In this context, this study aimed to understand the potential use of the CwPAMS app to guide antimicrobial prescribing, and specifically to serve as an educational tool for healthcare students in Commonwealth countries within the African continent. The study was also planned to evaluate knowledge and attitudes of healthcare students towards antimicrobial resistance and stewardship activities.

## 2. Materials and Methods

### 2.1. Survey Development

A survey- based approach was adopted, using a 59-item questionnaire (Supplementary Materials) developed from two existing validated surveys in the literature; European survey of healthcare workers’ knowledge, attitudes and behaviours with respect to antibiotics, antibiotic use and antibiotic resistance [15] and healthcare students’ survey assessing the knowledge, attitudes and behaviors of human and animal health students towards antibiotic use and resistance [16]. The survey was hosted on Survey Monkey, a web-based survey platform. A pilot survey was initially conducted in Nigeria and Uganda with seventeen [17] health care students. The survey was updated based on feedback and disseminated to healthcare students across ten African countries. Time taken to fill the survey and other suggestions for improvement were noted during the pilot. Pilot data was excluded from the final analysis. All questions were presented to respondents in English language. The questions were a mixture of Likert-scale, True or False, and choice format. The questionnaire included study information about respondents, inclusion of antimicrobial stewardship as part of their learning, existence of educational content focusing on AMR and stewardship, and explored perspectives to understand the potential benefits of a mobile application that guides antimicrobial prescribing. It also focused on information about individuals who had participated in a prudent antibiotic use campaign in the past three years. The survey was reviewed and refined by discussing with the CwPAMS project team and CPA’s global AMR lead. 

### 2.2. Target Respondents and Data Collection Procedure

The target population were undergraduate health care students from universities in Africa. Promotion was primarily to eight countries that are part of the original and extension phase of CwPAMS programme—Ghana, Kenya, Malawi, Nigeria, Uganda, Tanzania, Zambia. Healthcare students from diverse academic backgrounds were eligible to participate in this survey. Participation was voluntary, with the survey being open for responses over a 6-week period (12 April until 20 May 2021).

The survey was promoted through the support of CwPAMS representatives to health students in the selected African countries. The survey was also promoted through social media platforms amongst students and university leads. 

### 2.3. Data Analysis

Descriptive statistics on the frequency distributions and percentages were used to analyse the responses and IBM SPSS Statistics 25 and Microsoft Excel 2016 were used to analyse the data.

### 2.4. Ethics

Formal ethics approval was not required because this study was classified as a service evaluation of CPA’s activities. Informed consent was obtained from all the respondents to participate in the study (100%). No personal and identifiable data was collected and respondents were assured that the information provided would be kept confidential. Participation in this study was optional, and they had the option to withdraw their consent if they no longer agreed to any aspect. The data were held securely and in-line with the General Data Protection Regulation 2016/679.

## 3. Results

### 3.1. Demographics of Respondents and Countries Involved 

The study included responses from 483 health care students from 44 universities across 10 Commonwealth countries; Nigeria (313), Kenya (111), Uganda (34), Tanzania (16), Zambia (2), Malawi (2), India (1), Democratic Republic of Congo, Rwanda (1 student each). The respondents were studying a range of health related courses 250 pharmacy, 84 pharmacology, 66 medicine, 42 pharmacy technicians, 31 biomedical sciences, 5 nursing, 1 dentistry, 1 veterinary medicine and 3 others, which are part of the aforementioned courses). Most of the respondents had at least 4 years and above for their chosen undergraduate degree (90.2). The majority of respondents were already in their penultimate and final year of undergraduate degree; third year (23.6%) and fourth year of education (31.1%) (Table 1).

### 3.2. Knowledge of Antimicrobials, Antimicrobial Use and Stewardship 

#### 3.2.1. Teachings on Antibiotic Treatment and Usage

Most of the respondents reported that they received lectures about antibiotics during their undergraduate degree programme. More than two thirds of the respondents had teaching on prudent antibiotic use (70.4%), diagnosis of infections (74.3%), and antibiotic treatment (77.2%). Few were not taught about the prudent use of antibiotics (18.4%), diagnosis of infections (14.3%) and treatment with antibiotics (14.3%). Small percentages were not sure if they had been taught about the prudent use of antibiotics (11.2%), diagnosis of infections (11.4%) and treatment with antibiotics (8.5%) (Supplementary Material Table S1).

#### 3.2.2. Examination Questions on Antibiotic Use

Most of the respondents reported having had exam questions on antibiotics. More than half of them had exam questions on: prudent antibiotics (60.2%); diagnosis of infections (66.0%) and antibiotic treatment (70.0%). Only a few had no exam questions on prudent use of antibiotics (25.9%), diagnosis of infections (21.5%) and antibiotic treatment (18.8%). A small percentage was not sure if they had exam questions on prudent use of antibiotics (13.9%), diagnosis of infections (12.4%) and antibiotic treatment (11.2%) (Supplementary Material Table S2).

#### 3.2.3. Students’ Participation in Public Health Campaigns on Prudent Antibiotic Use 

Many of the respondents (69.6%) reported that they had not been involved in a campaign linked to prudent antibiotic use in the previous three years, 18% stated they had participated in campaigns and 12.4% could not remember whether they had participated or not. Of the 87 respondents that participated in a prudent antibiotic use campaign, 39.1% provided education on antimicrobial resistance, 20.7% reported they shared their knowledge about antimicrobial resistance on social media, others participated in Antimicrobial Resistance Seminar (10.3%), engaged in rallies and campaigns (9.2%), joined debates and conferences (4.6%), and (4.6%) helped to raise community awareness in a rally (Table 2). 

#### 3.2.4. Teaching Methods for Prudent Antibiotic Use

The most common method used for teaching the majority of the respondents on prudent antibiotic use was lecturing to more than 15 people (61.7%). Another teaching/learning technique frequently used was active learning tasks such as reading articles, group work, preparing an oral presentation (53.4%), e-learning (52.8%), discussion of clinical cases and vignettes (40.0%), peer or near-peer teachings such as classes led by other students or recently qualified doctors (39.3%), and digital resources such as apps (36.0%). On the other hand, unpopular learning methods included small group teachings with less than 15 people and clinical microbiology placement as reported by almost half of the respondents (Table 3).

#### 3.2.5. Reference Sources for Information on Antimicrobials 

Almost half of the students stated they did not know of any source of information on antimicrobials (47.0%) Those who had an antimicrobial reference source represented 40.8%, and 12.2% could not decide whether they knew or not. There were mixed responses on the reference sources that respondents were using with the highest proportion (13.5%) stating antimicrobial textbooks, followed by 8.9% who stated internet research and 7.2% who stated Emdex. Less than 5% referred to other sources including the BNF and the World Health Organization handbook on antimicrobial stewardship. Almost half (48.9%) did not specify the reference sources they currently use (Supplementary Material Table S3).

An online learning platform was the most popular reference source of information used by the students. Half of the students mainly used online libraries for their medical reference (48.0%), 35% used textbooks, 8% used the BNF and 9% used other sources. 

#### 3.2.6. Antimicrobial Stewardship Awareness amongst Students

Slightly more than half (52.2%) of participants reported that they had no prior knowledge of the term antimicrobial stewardship (AMS); 42% stated they had heard of AMS and 5.8% were unsure.

#### 3.2.7. Barriers to Up-to-Date Information about Drugs amongst Students 

About half of the respondents indicated that one of the challenges in obtaining up-to-date information on drugs was lack of resources (50.6%). Other factors highlighted included internet access (29.8%), power outages (17.9%), reliable sources (0.7%) and laxity (0.2%). Only 0.9% had no challenges in obtaining current information (Supplementary Material Table S4).

#### 3.2.8. Social Media Channels Used Mostly amongst Students

When asked “What social media channel do you think students relate with the most?”; the most common channels selected by the respondents were Twitter (33.5%), Instagram (23.6%), Facebook (17.2%) and WhatsApp (16.8%). Others included YouTube (0.4%), Zoom (0.2%), the Internet (0.2%) and Google (0.2%) (*n* = 483). There was no response from 6.8% of the respondents (Supplementary Material Table S5).

### 3.3. Students’ Perspectives on Antimicrobial Resistance and Stewardship

#### 3.3.1. Students’ Perspectives on Antibiotics Use

The majority (73.5%) of respondents believed that: unnecessary use of antibiotics makes them ineffective, that taking antibiotics is associated with side effects or risks, such as diarrhoea, colitis and allergies (66.7%), that healthy people can carry antibiotic-resistant bacteria (55.3%), and that any person treated with antibiotics is at increased risk of antibiotic-resistant infection (49.9%) (Table 4). About half of the respondents reported that antibiotics are not effective against viruses (53.6%), cold, and flu (42.4%). Approximately a quarter of the respondents (23.6%) could correctly answer that the use of antibiotics for growth promotion in farm animals is not legal in the EU.

More than three quarters of the students (82%) agreed or strongly agreed that prescribing, dispensing or administering inappropriate or unnecessary antibiotics was professionally unethical. A similar proportion of students strongly agreed or agreed that antibiotic resistance is both a national problem (73%) and one that would be a problem for their future individual practice (71%) (Table 5).

#### 3.3.2. Students’ Perspectives on Indiscriminate Antimicrobial Use in Respondents’ Countries

The majority of the respondents (74.9%) thought that the rate of the indiscriminate use of antimicrobials in their countries was alarming; 13% chose maybe, 6% respectively disagreed or were unsure.

#### 3.3.3. Students’ Perspectives on Education about Antimicrobial Resistance and Antimicrobial Stewardship

There were mixed responses to the statements measuring students’ perspectives on antimicrobial resistance and stewardship as shown in Table 6. More than a quarter (40.6%) of the respondents indicated that antimicrobial resistance (AMR) is a recurrent theme in their teaching and 40.8% reported that there is not enough educational content about AMR/AMS at their institution. More than half of the students thought that more AMR and stewardship learning content should be offered (56.1%) and 55.9% stated that an application that provides essential information about antimicrobials would be helpful for their learning. More than half also highlighted that increasing national campaigns on the prudent use of antimicrobials would help disseminate information about antimicrobial resistance and 54.7% agreed that social media would help increase awareness of antimicrobial stewardship. Those who would likely engage with an educational tool such as a phone app to learn more about antimicrobials accounted for 50.1%. Slightly more than half (52.1%) believed a medical information app offline and on the go will help them make more informed decisions about antibiotics. However, 52.2% of respondents believed antimicrobial resistance is not really as it seems, with new antibiotics being developed by scientists every year.

### 3.4. Factors Contributing to AMR

There was a mixed response on the highest implicating factor in antimicrobial resistance. Approximately one-third of respondents reported that the factor contributing most to AMR was not completing the full course of antibiotics (36.6%) or an inappropriate prescription of antibiotics (34.8%). Other respondents thought the highest implicating factor was poor management of antibiotic residues (9.3%), lack of infection control (5.6%), and poor hygiene (4.8%) (Supplementary Material Table S6).

### 3.5. Responsibility for Antimicrobial Stewardship

Most respondents stated that health professionals (61.8%) have the greatest responsibility for antimicrobial stewardship, followed by the Ministry of Health (26.5%), medical institutions (9.7%), all individuals (1.1%), the community (0.2%), and patients themselves (0.4%) (Supplementary Material Table S7).

### 3.6. Topics Students Would like more Information about and Educational Tools Students Would Use if Available

**Figure 1:**
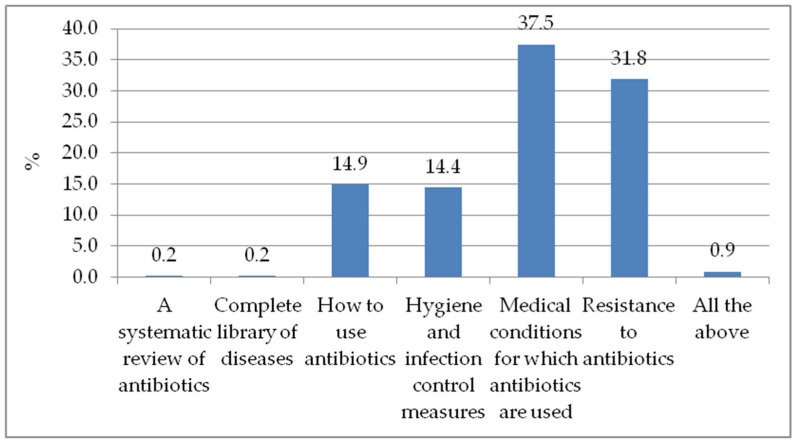
The topics on which to receive more information about.

There was a mixed response on the topics healthcare students would like to receive more information about (Figure 1). The medical conditions for which antibiotics are used ranks as the primary topic students want to know more about (37.5%). Others include resistance to antibiotics (31.8%), methods of antibiotic use (14.9%), hygiene and infection control measures (14.4%), and 0.2% each for a systematic review of antibiotics and a complete library of diseases. Of all respondents, less than 1% (0.9%) stated that they would like more information on all the topic options provided.

**Figure 2:**
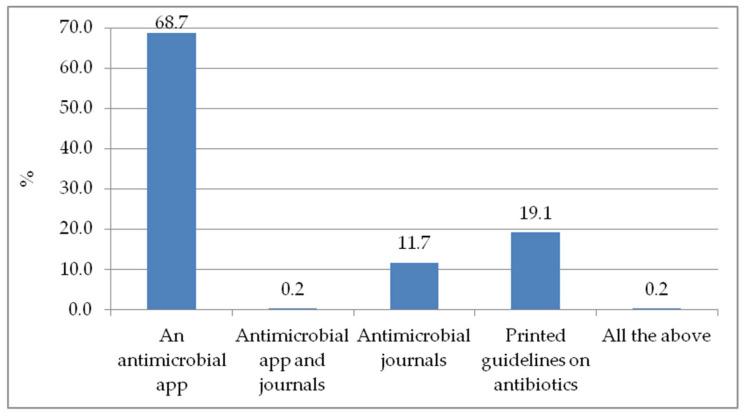
Educational tools that will be part of my regular learning, if available.

The majority of the respondents would like to use an antimicrobial app as an educational tool that is part of their regular learning, if available (68.7%). Printed guidelines on antibiotics would be an alternative for 19.1% of them, and antimicrobial journals were of interest to 11.7%. A combination of antimicrobial app and journals was found appealing by 0.2%, as shown in Figure 2.

## 4. Discussion

This is the first service assessment to consider the possibility of rolling out the CwPAMS Antimicrobial prescribing app to undergraduate students as an educational and learning tool. The study also aimed to assess the knowledge and perspectives of health care students towards AMR and stewardship.

### 4.1. Knowledge and Students’ Perceptions on Antimicrobial Stewardship

The study demonstrated that education on antimicrobials is highly considered in universities; more than 70% of the study respondents agreed that they had been taught about prudent use of antibiotics, diagnosis of infections and their management using antibiotics (Supplementary Material Table S1). This is important as, previously highlighted in a knowledge, attitude and practice study conducted among medical students in Colombia, university training improved knowledge of students on antimicrobials and their use [17]. In that study and a similar one conducted in China it was revealed that the knowledge level on antimicrobials increased as students progressed to higher academic years of study within their respective programmes [16,17,18].

Various educational strategies were used to educate students on prudent use of antimicrobials with the most common being lectures of at least 15 people (61.1%), oral presentation (53.4%) and e-learning (52.8%) (Table 3). These methods are not unique to the current study participants; the literature shows they have been used elsewhere, for example, in European settings, with lectures also being the most commonly used strategy [19,20]. 

While close to 70% of the respondents thought that a mobile application app would be essential in increasing their knowledge on the prudent use of antimicrobials, at the time the study was conducted, less than half (36.0%) of the participants previously had an opportunity to use digital resources such as apps for learning (Figure 2). As access to smart phones increases, several studies recommend the usage of mobile applications for medical education, as they have been shown to facilitate quick and timely access to information, flexible learning and peer interactions, thus improving efficiency and patient care [21,22,23,24]. Despite the advantages that come with using apps in patient care, some studies have noted that these can be sources of disruptions while providing patient care, as clinicians’ attention may be drawn to notifications from other personal mobile apps [22]. Furthermore, usage of phone could also be perceived negatively by patients as demonstrated in previous studies [13,25]. 

Earlier research findings have shown that usage of mobile applications in medical education can be facilitated by affordable cost and the ability to use the phone in an offline mode, thus not requiring internet connectivity and also reducing interference from other mobile apps [21,22]. Those facilitators were considered when developing the CwPAMS app as it is freely accessible without cost and, once downloaded, there is no need for internet connectivity to use it [13]. 

Despite this study reflecting a high consideration of prudent use of antimicrobials as part of the education system, the study also revealed notable gaps in the knowledge and access to information on antimicrobial usage. For instance, more than half of the participants had no prior knowledge on the term antimicrobial stewardship (AMS) and reported a lack of resources for obtaining up to date information on drugs (Supplementary Material Table S4). This is similar to a research finding among physicians in Nigerian Hospitals [26]. Relatedly, almost 70% of the study participants reported not participating in a campaign that included prudent antibiotic use in the previous three years (Table 2) and close to 50% of them did not know any source of information about antimicrobials. These findings indicate that besides the formal educational curriculum, there are opportunities for engaging and empowering students in Africa in antimicrobial stewardship. This is also evident in the study participants’ perspectives, where more than half of the students noted their need for more AMR and stewardship content, and suggested that increasing national campaigns on the prudent use of antimicrobials would help disseminate information about antimicrobial resistance. Previous studies also recommend the usage of various educational strategies to improve access to information on antimicrobial resistance and stewardship among students [27,28,29]. In that regard, interventions such as the CwPAMS app, which has been proven to provide easy access to infection management resources and to improve the appropriate use of antimicrobials according to national and international guidelines, can be an important tool in empowering students in Africa [13]. This study revealed that if an intervention like a prescribing app is availed, more than half of the students could potentially use it to as an educational tool (Figure 2). 

Additionally, 54.7% of the study participants thought that social media could help as part of improving their knowledge on antimicrobial resistance and stewardship. Here, the most preferred social media channels were Twitter, Instagram, Facebook and WhatsApp. With about 47% of the global population having access to the internet via mobile phones, the usage of social media has increased and this should be taken as an opportunity to increase awareness about disease prevention, and rational use of antibiotics and antibiotic resistance among the population and health workers [3,30,31]. An earlier research study about usage of social media in LMICs demonstrated that social media can be used as a platform to promote various educational interventions such as videos, games, and images [3]. As such, social media can be leveraged for promoting the usage of interventions such as the CwPAMS app that has proven to be an essential tool for enhancing antimicrobial stewardship in Africa. 

With most of the study respondents (61.8%) indicating that health professionals have the greatest responsibility for antimicrobial stewardship (Supplementary Material Table S6), it suggests that as health care students, they are aware that students have an important role to play. Thus, if empowered and given the opportunity, they can contribute to the reduction in the burden of AMR, as recommended in another study that increasing the knowledge of healthcare students, especially pharmacists who will have frequent interactions with antimicrobial users in the future, is key [32]. Students who participated in this study felt the need for more knowledge in some of the following areas: medical conditions for which antibiotics are used, resistance to antibiotics, methods of antibiotic use, hygiene, and infection control measures (Figure 1). The CwPAMS app can be an appropriate intervention for those needs as it hosts national and international guidelines with instructions on antimicrobial usage and infection prevention and control [13]. 

### 4.2. Strengths and Limitations

Although the survey includes responses from several respondents in Low-Middle-Income-Countries (LMIC) within the Commonwealth, the number of respondents for some countries was low, and only selected African countries participated in the survey. In addition, the number of medical students who responded to the survey was disproportionately lower than that of students from the pharmacy profession; however some of our findings such as knowledge of AMS amongst health students is similar to a study conducted amongst physicians in Nigeria, which reported that although physicians are familiar with AMR they have limited knowledge of AMS [26]. The results do not provide in-depth details of the issues within each country or across educational institutions, hence we cannot determine the local learning needs across regions. Further evaluation, perhaps using follow-up surveys, would be required to explore this. Nevertheless, the survey provides useful data on healthcare students’ knowledge and perspectives towards AMR and provides insights into the potential use of mobile applications such as the CwPAMS app for educating health students.

## 5. Conclusions

While progress has been made through collaborative programs to optimize the appropriate use of antimicrobials, more concerted effort is required to understand the needs of healthcare students and channel educational resources to improve their knowledge and involvement in antimicrobial stewardship. The study findings highlight that university education provides an avenue for health care students to learn about prudent antimicrobial usage. However, the study also revealed several opportunities for strengthening the knowledge and engagement of students in antimicrobial stewardship. The demonstration of poor knowledge of the term antimicrobial stewardship and limited access to related information shows that there could be value in the use of mobile applications such as CwPAMS app as an educational tool for AMR and AMS amongst healthcare students in LMICs. The strength of successful usage of the CwPAMS app lies in the fact that it can be used in an offline mode and is freely accessible. It is important that global health organisations such as the Commonwealth Pharmacists Association (CPA), leading on AMS interventions, consider expanding their support beyond qualified healthcare professionals to include the education and training of health care students; working in collaboration with international health student bodies such as the International Pharmaceutical Students’ Federation and International Federation of Medical Students’ Associations. This approach can potentially establish embedding use of evidence based guidelines in preparation for their professional careers.

## Figures and Tables

**Table 1:** Demographics of respondents and countries involved (across 10 Commonwealth countries).

Variables		Frequency	Percentage (%)
The undergraduate degree being studied	Pharmacy	250	51.8
	Pharmacology	84	17.4
	Medicine	66	13.7
	Pharmacy Technician	42	8.7
	Health sciences (biomedical sciences)	31	6.4
	Nursing	5	1
	Others (Veterinary Medicine, Dentistry, Clinical Pharmacology & Therapeutics, Clinical Medicine)	5	1
	**Total**	**483**	**100.0**
Years of the undergraduate degree for the chosen career	3 years	47	9.7
	4 years	158	32.7
	5 years	213	44.1
	6 years	6	1.2
	>6 years	59	12.2
	**Total**	**483**	**100.0**
Country	Nigeria	313	10.1
	Kenya	111	20.1
	Uganda	34	23.6
	Tanzania	16	31.1
	Ghana, Rwanda, Congo, Malawi, Zambia and Others	9	11.2
	**Total**	**483**	**100.0**

**Table 2:** Students’ participation in public health campaigns on prudent antibiotic use.

Variables		Frequency	Percentage (%)
**Participation in a campaign on the prudent use of antibiotics over the last three years**	Yes	87	18.0
	No	336	69.6
	Cannot remember	60	12.4
**Means of participation**	Through rallies and campaigns	8	9.2
	Surveys on AMR and Surveys on AMR and data collection (4.5%)	4	4.5
	Social media awareness on antimicrobial resistance	18	20.7
	SARS committee member MKU chapter	2	2.3
	Outreach program to secondary schools in Ilala Tanzania	2	2.3
	In debate and conferences	4	4.6
	I wrote a poem on antimicrobial resistance for the world health students’ alliance.	2	2.3
	Educating people about antimicrobial resistance	34	39.1
	Community awareness rally	4	4.6
	Attended an AMR seminar	9	10.3
	**Total**	**87**	**100.0**

**Table 3:** Teaching methods used to teach students about prudent use of antibiotics/antibiotic treatment.

Statements	Yes	No	Unsure
Lectures (with >15 people)	295 (61.1%)	95 (19.7%)	93 (19.3%)
Small group teaching (with <15 people)	96 (19.9%)	217 (44.9%)	170 (35.2%)
Discussions of clinical cases and vignettes	193 (40.0%)	143 (29.6%)	147 (30.4%)
Active learning assignments (e.g., article reading, group work, preparing an oral presentation)	258 (53.4%)	109 (22.6%)	116 (24.0%)
E-learning	255 (52.8%)	115 (23.8%)	113 (23.4%)
Role play or communication skills sessions dealing with patients demanding antibiotic training	147 (30.4%)	183 (37.9%)	153 (31.7%)
Infectious diseases clinical placement (i.e., clinical rotation or training in infectious diseases, involving patients)	131 (27.1%)	185 (38.3%)	131 (27.1%)
Microbiology clinical placement	117 (24.2%)	216 (44.7%)	150 (31.1%)
Peer or near peer-teaching (i.e., teaching led by other students or recently qualified doctors)	190 (39.3%)	159 (32.9%)	134 (27.7%)
Use of digital resources such as apps	174 (36.0%)	169 (35.0%)	140 (29.0%)

**Table 4:** Students’ responses to statements on antibiotics.

Statement (Correct Answers— False (F); True (T))	True	False	Unsure	Do Not Know
Antibiotics are effective against viruses (F)	118 (24.4%)	259 (53.6%)	81 (16.8%)	25 (5.2%)
Antibiotics are effective against cold and flu (F)	176 (36.4%)	205 (42.4%)	72 (14.9%)	30 (6.2%)
Unnecessary use of antibiotics makes them become ineffective (T)	355 (73.5%)	43 (8.9%)	56 (11.6%)	29 (6.0%)
Taking antibiotics have associated side effects or risks such as diarrhea, colitis, allergies (T)	322 (66.7%)	39 (8.1%)	90 (18.6%)	32 (6.6%)
Every person treated with antibiotics is at an increased risk of antibiotic-resistant infection (T)	241 (49.9%)	111 (23.0%)	91 (18.8%)	40 (8.3%)
Bacteria that are resistant to antibiotics spread easily from person to person (T)	224 (46.4%)	95 (19.7%)	118 (24.4%)	46 (9.5%)
Healthy people can carry antibiotic resistant bacteria (T)	267 (55.3%)	68 (14.1%)	101 (20.9)	47 (9.7%)
The use of antibiotics to stimulate growth in farm animals is legal in the EU (F)	114 (23.6%)	69 (14.3%)	216 (44.7%)	84 (17.4%)

**Table 5:** Level of agreement or disagreement with antibiotic prescribing patterns.

Statements	Strongly agree	Agree	Neutral	Strongly Disagree	Disagree	No Response
Most coughs, colds and sore throats get better on their own without the need for antibiotics	162 (33.5%)	156 (32.3%)	85 (17.6%)	13 (2.7%)	23 (4.8%)	44 (9.1%)
Prescribing, dispensing, or administering inappropriate or unnecessary antibiotics is professionally unethical	315 (65.2%)	81 (16.8%)	26 (5.4%)	7 (1.4%)	7 (1.4%)	47 (9.7%)
There are enough antibiotics under development worldwide to keep up with the problem of resistance	99 (20.5%)	103 (21.3%)	116 (24.0%)	49 (10.1%)	67 (13.9%)	49 (10.1%)
Antibiotic resistance is a national problem	228 (47.2)	128 (26.5%)	60 (12.4%)	9 (1.9%)	9 (1.9%)	49 (10.1%)
I think the antibiotic resistance will be a problem for my future individual practice	240 (49.7%)	100 (20.7%)	63 (13.0%)	9 (1.9%)	19 (3.9%)	52 (10.8%)

**Table 6:** Students’ perspectives on antimicrobial resistance and stewardship.

Variables	Strongly Agree	Agree	Neutral	Strongly Disagree	Disagree	No Response
Antimicrobial resistance (AMR) is a recurring theme in my learning	71 (14.7%)	126 (26.1)	97 (20.1)	94 (19.5)	50 (10.4)	45 (9.3%)
There is enough educational content around AMR/AMS in my institution	24 (5.0%)	84 (17.4%)	129 (26.7%)	61 (12.6%)	136 (28.2%)	49 (10.1%)
I think more learning content about AMR and stewardship opportunities should be made available	172 35.6%)	99 (20.5%)	43 (8.9%)	108 (22.4%)	18 (3.7%)	43 (8.9%)
An App that provides important information about antimicrobials will be useful for my personal learning	172 (35.6%)	98 (20.3%)	45 (9.3%)	106 (21.9%)	20 (4.1%)	42 (8.7%)
Reinforcement of national campaigns around prudent antimicrobial use will help spread information about antibiotic resistance	155 (32.1%)	110 (22.8%)	47 (9.7%)	106 (21.9%)	17 (3.5%)	48 (9.9)
Social media handles among student health professionals will help to raise more awareness of antimicrobial stewardship.	138 (28.6%)	126 (26.1%)	53 (11.0%)	91 (18.8%)	25 (5.2%)	50 (10.4%)
Antimicrobial resistance is not really as it seems because new antibiotics are developed yearly by scientists	26 (5.4)	47 (9.7%)	99 (20.5%)	125 (25.9%)	127 (26.3%)	59 (12.2%)
I am likely to engage with an educational tool such as an App on my phone to learn about antimicrobials	117 (24.2%)	125 (25.9%)	71 (14.7%)	90 (18.6%)	23 (4.8%)	57 (11.8%)
Having an offline and on-the-go medical information app will help me make more informed choices about antibiotics	147 (30.4%)	105 (21.7%)	55 (11.4%)	102 (21.1%)	20 (4.1%)	54 (11.2%)

## Data Availability

Data is contained within the article or Supplementary Material.

## Supplementary Materials

The following supporting information can be downloaded at: https://www.mdpi.com/article/10.3390/antibiotics11050691/s1, Table S1: Teaching about antibiotic treatment and prudent antibiotic use during my studies; Table S2: My examinations included questions on antibiotic treatment or prudent use of antibiotics; Table S3: Reference sources being used; Table S4: Challenges encountered in obtaining current information about drugs; Table S5: Social media channels students relate with the most; Table S6: The highest implicating factor in AMR; Table S7: Who has the highest responsibility for antimicrobial stewardship?.
