# Supplementary material for: Exploring the Antimicrobial Stewardship Educational Needs of Healthcare Students and the Potential of an Antimicrobial Prescribing App as an Educational Tool in Selected African Countries
Source: Antibiotics (Basel). 2022 May 19;11(5):691. doi: 10.3390/antibiotics11050691 (PMC9137764; doi:10.3390/antibiotics11050691)
Supplement: Supplementary file 1 [file antibiotics-11-00691-s001.zip › antibiotics-1695351-supple.pdf]

Supplementary Materials

Table S1. Teaching about antibiotic treatment and prudent antibiotic use during my studies.

Variables	Yes	No	Unsure	Total
Prudent antibiotic use	340(70.4%)	89(18.4%)	54 (11.2%)	*483(100)*
Diagnosis of infections	359(74.3%)	69(14.3%)	55(11.4%)	*483(100)*
Antibiotic treatment	373 (77.2%)	69(14.3%)	41(8.5%)	*483(100)*

Table S2. My examinations included questions on antibiotic treatment or prudent use of antibiotics.

Variables	Yes	No	Unsure	Total
Prudent antibiotic use	291 (60.2%)	125 (25.9%)	67 (13.9%)	*483(100)*
Diagnosis of infections	319 (66.0%)	104 (21.5%)	60(12.4%)	*483(100)*
Antibiotic treatment	338 (70.0%)	91(18.8%)	54(11.2%)	*483(100)*

Table S3. Reference sources being used.

Sources of reference	*Frequency*	*Percentage (%)*
Textbooks	65	13.5
Internet Search	43	8.9
Emdex	35	7.2
Not sure of any	22	4.3
Medscape	17	3.5
BNF	10	2.1
Standard Treatment Guideline	7	1.4
Textbooks and Internet	6	1.2
World Health Organization handbook on Antimicrobial Stewardship	5	1
Drug Index	4	0.8
Lecture notes and Internet	4	0.8
Lecture notes and textbooks	3	0.6
Uganda Clinical Guidelines	3	0.6
Internet and Textbooks	2	0.4
Kaplan Medical Teachings	2	0.4
Others (apps, articles & lectures, Aulton Pharmaceutics, British National Formulary, Clinical pharmacy by Robert Walker, Future Learn, Global Health Antimicrobial Resistance Platform, Google Scholar & School Library, Handouts given by lecturer, Lab Manuals, Lippincott, Mdex, My father is a certified pharmacist, National Multisectoral Antimicrobial Resistance Platform, Newspaper, PubMed journal articles, Tanzania Treatment Guideline, Textbook & Research Methods, Tripathi)	19	3.8
No response	236	48.9
Total	***483***	***100.0***

Table S4. Challenges encountered in obtaining current information about drugs.

Statements	Frequency	Percentage (%)
Lack of resources	226	50.6
Internet access	133	29.8
Power Outage	80	17.9
Laxity	2	0.2
None	4	0.9
Reliable sources	2	0.7
Total	447	100.0
No response	36	-

Table S5. Social media channels students relate with the most.

Statements	Frequency	Percentage (%)
Twitter	162	33.5
Instagram	114	23.6
Facebook	83	17.2
WhatsApp	81	16.8
YouTube	2	0.4
Others	8	1.6
No response	33	6.8
**Total**	**483**	**100.0**

Table S6. The highest implicating factor in AMR.

Statements	Frequency	Percentage (%)
Not completing a full course of antibiotics	174	36.6
Inappropriate antibiotic prescribing	168	34.8
Improper management of left-over antibiotics	45	9.3
Lack of infection control	27	5.6
Poor hygiene	23	4.8
All the above	2	0.4
Others	3	0.6
No response	41	8.5
Total	**483**	**100.0**

Table S7. Who has the highest responsibility for antimicrobial stewardship?

Variables	Frequency	Percentage (%)
Healthcare professionals	275	61.8
Ministry of health	118	26.5
Medical institutions	43	9.7
Everyone	5	1.1
Patients	2	0.4
All the above	1	0.2
Community education	1	0.2
Total	445	100.0
No response	38	-
