# Supplementary material for: Exploring the Antimicrobial Stewardship Educational Needs of Healthcare Students and the Potential of an Antimicrobial Prescribing App as an Educational Tool in Selected African Countries
Source: Antibiotics (Basel). 2022 May 19;11(5):691. doi: 10.3390/antibiotics11050691 (PMC9137764; doi:10.3390/antibiotics11050691)
Supplement: Supplementary file 1 [file antibiotics-11-00691-s001.zip › Ogunnigbo et al_Exploring the antimicrobial stewardship educational needs_Health Students Survey.pdf]

Final-CwPAMS App Student Survey

The Commonwealth Partnerships for Antimicrobial Stewardship (CwPAMS) App was launched in 2020 by the Commonwealth Pharmacist Association. Aim of the app is to provide antimicrobial prescribing guidelines and serve as an educational tool for prescribers, pharmacists, nurses, dentists and other healthcare professionals.

Healthcare students in Nigeria, Uganda, Tanzania, Zambia, Malawi, Kenya, Zambia and Sierra Leone are being invited to participate in a survey in order to understand possibilities for use of the app by health students

Participation is voluntary. All data collected will be stored securely and anonymously, in line with best practice, data protection and confidentiality guidelines. Where there are any publications as a result of the survey, they will be in combination with other questionnaires completed by healthcare students in other Commonwealth countries. Deadline for completion - 20th May 2021

diane.ashiru-oredope@commonwealthpharmacy.org (not shared) Switch account

* Required

I give my consent to contribute to this study *

- Yes
- No

What career are you currently studying for? *

- Medicine
- Pharmacy
- Nursing
- Dentistry
- Health sciences e.g. biomedical sciences
- Other:

How many years is the undergraduate degree for your chosen career? *

- 3 years
- 4 years
- 5 years
- 6 years
- > 6 years
- Other:

Year of Study *

- First year
- Second year
- Third year
- Fourth year
- Fifth year
- Other:

What is the name of your university *

Your answer

What country is your university in? *

- Ghana
- Uganda
- Tanzania
- Zambia
- Kenya
- Sierra Leone
- Malawi
- Nigeria
- Other:

Have you heard about antimicrobial stewardship (AMS) before now?

- Yes
- No
- Unsure

Have you had any teaching about antibiotic treatment and prudent antibiotic use during your undergraduate degree?

	Yes	No	Unsure
Prudent antibiotic use	○	○	○
Management of infections (diagnosis of infections)	○	○	○
Management of infections (antibiotic treatment)	○	○	○

Have any of your examinations included questions about antibiotic treatment or prudent use of antibiotics?

	Yes	No	Unsure
Prudent antibiotic use	○	○	○
Management of infections (diagnosis of infections)	○	○	○
Management of infections (antibiotic treatment)	○	○	○

Which of the following methods of teaching have been used to teach you about prudent use of antibiotics/antibiotic treatment and how useful would you rate them?

	Yes	No	Unsure
Lectures (with >15 people)	○	○	○
Small group teaching (with <15 people)	○	○	○
Discussions of clinical cases and vignettes	○	○	○
Active learning assignments (e.g. article reading, group work, preparing an oral presentation)	○	○	○
E-learning	○	○	○
Role play or communication skills sessions dealing with patients demanding antibiotic training	○	○	○
Infectious diseases clinical placement (i.e. clinical rotation or training in infectious diseases, involving patients)	○	○	○
Microbiology clinical placement	○	○	○
Peer or near peer-teaching (i.e.teaching led by other students or recently qualified doctors)	○	○	○
Use of digital resources such as	○	○	○

apps

Do you think the rate of indiscriminate antimicrobial use in your country is alarming?

- Yes
- No
- Maybe
- Unsure

Do you know any reference source for information on antimicrobials?

- Yes
- No
- Maybe

What are the reference sources that you currently use?

Your answer

How much do you agree with the following statements

	Strongly disagree	Disagree	Neutral	Agree	Strongly agree
Antimicrobial resistance (AMR) is a recurring theme in your learning ?	○	○	○	○	○
There is enough educational content around AMR/AMS in your institution	○	○	○	○	○
Do you think more learning content about AMR and stewardship opportunities should be made available?	○	○	○	○	○
An App that provides important information about antimicrobials will be useful for your personal learning?	○	○	○	○	○
Reinforcement of national campaigns around prudent antimicrobial use will help spread information about antibiotic resistance	○	○	○	○	○
Will social media handles among student	○	○	○	○	○

health professionals help to raise more awareness of antimicrobial stewardship?					
Antimicrobial resistance is not really as it seems because new antibiotics are developed yearly by scientists	◯	◯	◯	◯	◯
I am likely to engage with an educational tool such as an App on my phone to learn about antimicrobials	◯	◯	◯	◯	◯
Having an offline and on-the-go medical information app will help me make more informed choices about antibiotics	◯	◯	◯	◯	◯

What sources of medical reference do you use the most for information?

- ◯ BNF
- ◯ Textbooks
- ◯ Online libraries
- ◯ Other

What challenges do you face in getting up-to-date drug information as a student?

- Internet access
- Lack of resources
- Power Outage
- Other:

What social media channel do you think students relate with the most?

- Instagram
- Twitter
- Facebook
- Other:

Which of the following do you think is the highest implicating factor in AMR?

- Not completing a full course of antibiotics
- Improper management of left over antibiotics
- Inappropriate antibiotic prescribing
- Poor hygiene
- Lack of infection control
- Other:

Who do you think has the greatest responsibility for antimicrobial stewardship?

- Ministry of health
- Medical institutions
- Healthcare professionals
- Other:

On which of the following topics, if any would you like to receive more information?

- How to use antibiotics
- Resistance to antibiotics
- Medical conditions for which antibiotics are used
- Hygiene and infection control measures
- Other:

Which of these educational tools will be a part of your routine learning if available?

- Printed guidelines on antibiotics
- An antimicrobial app
- Antimicrobial journals
- Other:

Answer whether you believe these statements are true or false.

	True	False	Unsure	Do not know
Antibiotics are effective against viruses	○	○	○	○
Antibiotics are effective against cold and flu	○	○	○	○
Unnecessary use of antibiotics make them become ineffective	○	○	○	○
Taking antibiotics has associated side effects or risks such as diarrhoea, colitis, allergies	○	○	○	○
Every person treated with antibiotics is at an increased risk of antibiotic resistant infection	○	○	○	○
Bacteria that are resistant to antibiotics spread easily from person to person	○	○	○	○
Healthy people can carry antibiotic resistant bacteria	○	○	○	○
The use of antibiotics to stimulate growth	○	○	○	○

in farm animals
is legal in the EU

How strongly do you agree or disagree with the following?

	Strongly agree	Agree	Neutral	Disagree	Strongly Disagree
Most coughs, colds and sore throats get better on their own without the need for antibiotics	○	○	○	○	○
Prescribing, dispensing or administering inappropriate or unnecessary antibiotics is professionally unethical	○	○	○	○	○
There are enough antibiotics under development worldwide to keep up with the problem of resistance	○	○	○	○	○
Antibiotic resistance is a national problem	○	○	○	○	○
Do you think antibiotic resistance will be a problem for your future individual practice?	○	○	○	○	○

Within the last 3 years have you been involved in a campaign linked to prudent use of antibiotics?

- ◯ Yes
- ◯ No
- ◯ Cannot remember

Please provide further details of how you were involved

Your answer

Approximately how long did it take you to complete the survey

- ◯ <5mins
- ◯ 6-10mins
- ◯ 11-15mins
- ◯ >15mins

Please use this space to provide any other relevant comments or feedback

Your answer

Submit Clear form

Never submit passwords through Google Forms.

This form was created inside of Commonwealth Pharmacists Association. Report Abuse

Google Forms
